# The Clock Counts – Length Effects in English Dyslexic Readers

**DOI:** 10.3389/fpsyg.2019.02495

**Published:** 2019-11-12

**Authors:** S. Provazza, D. Giofrè, A.-M. Adams, D. J. Roberts

**Affiliations:** ^1^School of Natural Sciences and Psychology, Liverpool John Moores University, Liverpool, United Kingdom; ^2^Department of Education Sciences, School of Social Sciences, University of Genoa, Genoa, Italy; ^3^Division of Psychology, Centre for Cognitive Neuroscience, College of Health and Life Sciences, Brunel University London, Uxbridge, United Kingdom

**Keywords:** developmental dyslexia, word length effect, dual-route model, triangle model, orthography, triangle model of reading, reading, dyslexia

## Abstract

In reading, length effects (LEs) are defined as an increment in the time taken to read as a function of word length and may indicate whether reading is proceeding in an efficient whole word fashion or by serial letter processing. LEs are generally considered to be a pathognomonic symptom of developmental dyslexia (DD) and predominantly have been investigated in transparent orthographies where reading impairment is characterized as slow and effortful. In the present study a sample of 18 adult participants with DD were compared to a matched sample of typical developing readers to investigate whether the LE is a critical aspect of DD in an opaque orthography, English. We expected that the DD group would present with marked LEs, in both words and non-words, compared to typical developing readers. The presence of LEs in the DD group confirmed our prediction. These effects were particularly strong in low frequency words and in non-words, as observed in reading speed. These preliminary findings may have important theoretical implications for current understanding of DD.

## Introduction

Developmental dyslexia (DD) is a specific learning disorder characterized by problems with accurate or fluent word recognition, poor letter decoding, and poor spelling abilities, that affects up to 15% of the population worldwide (American Psychiatric Association [APS], 2013). Although most of the research regarding DD has been conducted with children, reading difficulties persist throughout life (Bruck, 1985; Finucci et al., 1985; Nergård-Nilssen and Hulme, 2014; Shrewsbury, 2016; Eloranta et al., 2018).

The manifestation of DD differs across orthographies. For instance, in transparent orthographies in which the mapping between letters and sounds is more regular and predictable (e.g., Italian), the consistency of the letter-sound correspondence limits the incidence of letter decoding errors [e.g., *volpe* (fox) and read as *folpe*]. The main feature of DD in transparent orthographies appears to be slow and effortful word reading, with accuracy being relatively well preserved (Job et al., 1984; Wimmer, 1993; de Jong and van der Leij, 2003). Conversely, in opaque orthographies with more irregular letter-sound correspondence in which the mapping between letters and sounds is not always consistent and predictable (e.g., English), DD tends to be characterized by slow reading and a dramatic impairment in reading accuracy (Wimmer, 1993; Landerl et al., 1997; Spinelli et al., 2005). These patterns led (Wimmer, 1993) to propose a distinction between “speed dyslexia,” affecting individuals reading transparent orthographies, and “decoding dyslexia,” affecting individuals reading opaque orthographies (although see Ziegler et al., 2003 for similarities between accuracy and speed across orthographies).

Differences in the manifestation of DD in opaque and transparent orthographies might reflect variances in how reading is accomplished. Opaque orthographies encourage a whole-word reading procedure, due to orthographic irregularity (Frost et al., 1987; Marinelli et al., 2016; see also Ziegler and Goswami, 2005 for a review on differences between languages). Given the inconsistency of the mapping between letters and sounds, DD in opaque orthographies is characterized by a high incidence of errors (Wimmer, 1993). Conversely, transparent orthographies encourage a serial analysis of the word, particularly in the early stages of reading acquisition, due to the almost perfect concordance between the letters (graphemes) and the sounds (phonemes) of the words (Frost et al., 1987; Ziegler and Goswami, 2005). Given this letter-sound consistency, in transparent orthographies DD is mainly characterized by slow, although accurate reading (Wimmer, 1993; Coltheart and Leahy, 1996; Zoccolotti et al., 1999; Ziegler and Goswami, 2005; Martens and de Jong, 2006). This pattern of difficulties seems to persist in adulthood (Martin et al., 2010; Lindgrén and Laine, 2011; Re et al., 2011; Suárez-Coalla and Cuetos, 2015; Eloranta et al., 2018).

A cross-cultural study conducted with English and Italian children to investigate reading acquisition in these orthographies showed that, even in the early stage of reading acquisition, English children were faster than Italian children, although less accurate (Marinelli et al., 2016). Interestingly, a length effect (LE) was present in younger children in both groups, however, it disappeared in older English children and persisted only in Italian children. These results suggest that children reading a transparent orthography persisted in adopting a serial strategy, whilst children reading the opaque orthography did not. This pattern is consistent with evidence from adult English readers where exposure to words through reading acquisition decreases the likelihood that a serial, phonological decoding strategy will be employed. Given the characterization of reading impairment in transparent orthographies is captured in reading latency, the LE in DD has been more extensively evaluated in these orthographies in both adults and children (see Davies et al., 2007 for Spanish children; Richlan et al., 2010 for German adults; Suárez-Coalla and Cuetos, 2015 for spanish adults; Zoccolotti et al., 2005 for Italian children), but scarcely investigated in English (see e.g., Ziegler et al., 2003; Kemp et al., 2009).

Length effects have been considered as a pathognomonic symptom in acquired disorders of reading such as pure alexia (Behrmann and Shallice, 1995; Behrmann et al., 1998; Montant and Behrmann, 2001; Roberts et al., 2010, 2013, 2015), a disorder caused by damage to the left fusiform gyrus in the ventral occipitotemporal cortex (Price and Devlin, 2011; Behrmann and Plaut, 2013; Roberts et al., 2013). Support for the contention that this area may also be important in DD is provided by Richlan et al. (2010). They found that adult participants with DD presented with abnormalities of the left occipitotemporal cortex. In addition, reading performance of these participants was also captured by strong LEs. It should be acknowledged, however, that this evidence is from readers of a transparent orthography (German). Whether LEs are a core deficit in adult DD participants reading an opaque orthography is yet to be determined.

One cognitive model employed to explain the LE in reading is the dual-route cascaded (DRC) model (Coltheart et al., 2001). Although the DRC model was initially implemented to explain deficits in acquired dyslexia, it also accommodates deficits in developmental reading disorders and is widely employed in research on DD (Castles and Coltheart, 1993; Coltheart and Leahy, 1996; Castles et al., 2006; Coltheart, 2015).

In this model, reading can be achieved via two routes: (i) lexically through access to stored representations in the orthographic and phonological lexicons, and (ii) sub-lexically through a phonological conversion procedure. The lexical route permits reading of familiar words in parallel whilst the sub-lexical route processes unfamiliar words and phonologically plausible non-words (e.g., plur) through a serial spelling-to-sound (grapheme-to-phoneme) mechanism. In this conceptualization, the serial processing of graphemes results in a LE whereas words read via the lexical route, with parallel processing of graphemes, and predicts that a LE will not be observed. The larger the LE the greater the reliance on the sub-lexical route (Martens and de Jong, 2006). Hence, within the DRC model, the LE might be considered to reflect an over-reliance on the sub-lexical route (Barca et al., 2006).

An alternative to the DRC account of the underpinnings of reading achievement is the triangle model, which is implemented in a parallel distributed processing (PDP) connectionist network (Plaut et al., 1996). The triangle model has received substantial support in explaining various types of acquired dyslexia (Patterson and Lambon Ralph, 1999; Hoffman et al., 2015). This view differs from the DRC in that reading is underpinned by the phylogenetically more mature primary systems of vision, phonology, and semantics. Central to this approach is the proposal that the same computational elements, in various combinations, support different activities during word reading: (1) vision, which with respect to reading mediates knowledge about orthographic word form; (2) phonology – the internal representation of word sound; and (3) semantics – word meaning. Reading aloud can be accomplished directly between vision and phonology (V > P) or mediated by semantics (V > S or the interplay between S <> P). During reading acquisition, the direct pathway becomes sensitive to the relationship that exists between graphemes and phonemes and achieves efficient computations for regular words and non-words with typical grapheme-phoneme rules (e.g., *pat* and *snat*). It is less efficient for infrequent irregular words with atypical grapheme-phoneme rules (e.g., *poignant*) and it is these that may require additional semantic support. In the scenario of the triangle model, LEs may be the result of damage to the visual system (e.g., Roberts et al., 2013).

The present study aimed to examine whether LEs are present in DD reading of English orthography. Few studies have investigated LEs in English children with DD (for an exception see Ziegler et al., 2003) and to the best of our knowledge, evidence of LEs in adult English speakers with DD is scarce. It is possible that, even if LEs affect the reading performance of English children with DD, by adulthood they will have acquired adequate strategies to compensate for their deficit. However, it is also possible that the LEs persist in adulthood, suggesting an over-reliance on the sub-lexical route to read, in the scenario of the DRC model, or a deficit in the visual system, in the scenario of the triangle model. To evaluate between these possibilities, we compared a group of English university students with a diagnosis of DD, alongside a group of typically developing readers (TDR) in a word reading task. Such a population represents individuals who might have compensated their reading difficulties in some way and achieve well academically (Lefly and Pennington, 1991; Kemp et al., 2009; Cavalli et al., 2017). To do so they may have received extensive instructional support. Evidence from this population of a resistant LE therefore speaks to a more stringent test of a core deficit in reading processes. Both accuracy and reaction times (RTs) have been analyzed. Following evidence of increased reliance on the sub-lexical route with decreasing word familiarity (Weekes, 1997; Balota et al., 2004) both non-word reading and the effect of word frequency were also explored.

## Materials and Methods

### Participants

Eighteen university students with DD (5 males; age range 19–27; *M*_years_ = 21.8; *SD* = 2.29) participated. All had normal or corrected-to-normal vision and were in receipt of a formal diagnosis of dyslexia (supplied by a registered assessor of SpLD) as required for access arrangements and additional support in UK higher education institutions. These diagnoses follow DSM-IV recommendations (American Psychiatric Association [APS], 1994) and the guidelines adopted in public services, namely normal level of general intelligence (IQ above 85; although we did not obtain a measure of IQ as part of this study), reading performance at a clinical level, and no neurological, sensory, or educational deficit that could be cause of their reading impairment. They have been contrasted to a TDR group of 18 students (7 males; age range 19–28; *M*_years_ = 21.8; *SD* = 2). The two groups did not differ for gender [χ*^2^*(1) = 0.50, *p* = 0.480, Cramer’s *V* = 0.118] or age [*F*(1,34) = 0.02, *p* = 0.878, η*^2^_*p*_* = 0.001]. The study was reviewed and approved by the Liverpool John Moores University Research Committee and by the RES Committee North West Liverpool Central (15/NW/0461). Written consent was obtained from all participants.

### Materials and Procedure

#### Single Word Reading (Roberts et al., 2010)

In this and all subsequent tasks, stimuli were presented using E-Prime 2.0 software on a PC. Participants were seated approximately 50 cm from the screen. A list of 180 words comprising 60 words of three, five and seven letters were administered. These included 30 low frequency words and 30 high frequency words in each length set matched for CELEX written word frequency across the three letter lengths (three letters: low 1.08, high 151.96, average 76.52; five letters: low 1.10, high 130.76, average 65.93; seven letters: low 1.9, high 145.19, average 73.57 – for details see Roberts et al., 2010). Significant frequency effects were observed within each length and collapsed across length (*ts* > 6.8; *ps* < 0.001).

Stimuli were randomize and presented in the same order for each participant. Each word was presented after a fixation point with a duration of 500 ms, remaining on screen until the participant responded. Participants were instructed to read the words aloud as fast and accurately as possible. Reading latencies were measured using the E-Prime voice key and calculated from the onset of the stimulus to the onset of the correct naming response and, therefore, encompass the time taken to identify individual letters. Reading accuracy was recorded by the experimenter using a response box. Participant responses were also recorded allowing the accuracy of pronunciation to be agreed by two researchers. A number of responses were excluded from the analyses of RTs: incorrect responses, responses below 200 ms and those considered invalid due to technical problems (e.g., microphone errors).

#### Single Non-word Reading (Roberts et al., 2013)

Monosyllabic non-words of three, four, five, and six letters were used (17 for each length). Non-words were pronounceable letter strings, derived by changing one letter of a standardized English word list (Weekes, 1997, Roberts et al., 2013) and provided the initial phoneme of that word remained intact. Non-words were matched for number of phonemes, summed bigram frequency, and average grapheme frequency. The procedure was identical to that described above. It is important to note that the time between the onset of the word or non-word stimulus to the onset of the correct naming response is an indicator of the LE. Of course, when subjects begin to pronounce the string, they have already decided that reading is lexical or non-lexical.

#### Data Analytic Strategy

Generalized linear mixed-effects model (GLMM), a robust analysis that allows controlling for the variability of items and subjects (Baayen et al., 2002), was implemented. GLMM limits the loss of information due to the prior averaging of the by-item and by-subject analyses and has been repeatedly used in the case of RTs and errors (Paizi et al., 2013; Marinelli et al., 2016). Analyses were carried out by using R (R Core Team, 2019), with the package lme4 for fitting the models (Bates et al., 2015), and the package ggplot2 for the graphics (Wickham, 2009). The package lmerTest was used to obtain *p*-values and summary tables for lmer model fits on RTs (Kuznetsova et al., 2017), while a traditional model comparison was used for the accuracy. Participants and items were used as independent random effects. Fixed effects varied in different analyses.

As for words, Group (DD vs. TDR), Frequency (High vs. Low), and Length (3, 5, and 7 letters) were used as fixed factors. Concerning non-words, Group (DD vs. TDR), and Length (3, 4, 5, and 6 letters) were included as fixed factors. Analysis on the RTs were repeated using data transformation in *z*-scores, to control for over-additive effects (see Paizi et al., 2013 for a similar approach). It is worth noting that this transformation fixes the grand average of each participant (and therefore of each group) to zero. Therefore, in all *z*-score analyses the fixed effect of group and the random effects of subject tend to be closed to zero. Note that the higher the *z*-score, the lower the performance.

## Results

### *A priori* Power Analysis

Given the relatively small sample size a power analysis, using G-Power (Erdfelder et al., 1996) has been performed prior to data collection to determine the sufficiency of the sample estimating a moderate effect size based on Cohen’s (1988) thresholds. Considering an alpha level of 0.05, and a correlation between measurements of 0.05 a sample of 10 participants has a power of 0.80 to detect a significant interaction. Considering within factors effects, a sample size of 8–10 is required to detect significant differences with a power of 0.80. Finally, concerning the between factor effect, a sample of 28 is needed to have a power of 0.80 to detect significant effects. The sample size of 36, which was the sample size that we decided to obtain, has a power of 0.90 to detect a significant effect of the between factor manipulation. The analytic approach that we decided to use (i.e., GLMM), strengthen the experimental power of the by-subject and by-item analyses and limits the loss of information due to the prior averaging of the by-item and by-subject analyses (Baayen et al., 2002; Paizi et al., 2013).

### Descriptive Statistics

Means and standard deviations for both RTs and accuracy of the two groups are displayed in Table 1.

**TABLE 1:** Descriptive statistics for reading speed and accuracy as a function of group.

	**DD**		**TDR**		**Cohen’s *d***
	
**Measure**	***M***	***SD***	***M***	***SD***	
***Length RTs* (ms)**					
Word 3 letters	703.96	153.07	553.66	72.59	1.25
Word 5 letters	751.8	209.06	559.71	71.95	1.23
Word 7 letters	846.4	250.41	568.71	66.76	1.51
NW 3 letters	853.86	335.17	587.76	80.79	1.09
NW 4 letters	936.09	355.84	609.59	110.02	1.24
NW 5 letters	1084.44	496.72	620.01	114.88	1.29
NW 6 letters	1176.076	592.01	628.56	116.33	1.28
***Length accuracy* (%)**					
Word 3 letters	95	4	97	2	0.63
Word 5 letters	91	6	95	2	0.89
Word 7 letters	90	8	95	3	0.83
NW 3 letters	87	14	95	4	0.78
NW 4 letters	87	14	95	4	0.78
NW 5 letters	82	18	95	4	1
NW 6 letters	87	13	96	6	0.89
***Frequency RTs* (ms)**					
HF 3 letters	665.90	143.22	544.02	75.07	1.06
HF 5 letters	696.04	177.13	552.95	74.84	1.05
HF 7 letters	729.78	206.74	545.23	65.83	1.20
LF 3 letters	744.78	169.93	563.78	74.80	1.38
LF 5 letters	819.58	262.71	567.83	76.67	1.30
LF 7 letters	998.56	358.18	595.54	74.20	
***Frequency accuracy* (%)**					
HF 3 letters	98	1	98	1	0
HF 5 letters	97	3	99	1	0.89
HF 7 letters	98	2	99	1	0.63
LF 3 letters	92	8	96	3	0.66
LF 5 letters	85	9	92	6	0.91
LF 7 letters	83	15	91	7	0.68

*TDR, typically developing readers; DD, developmental dyslexics; HF, high frequency; LF, low frequency; NW, non-words; and RTs, reaction times in milliseconds (ms).*

### Word Reading

#### Reaction Times

**FIGURE 1:**
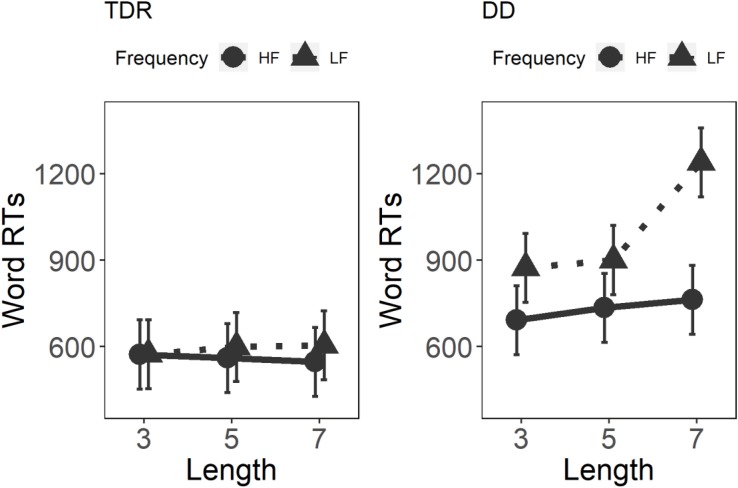
Three-way interaction on the speed on Words. TDR, typical developing readers; DD, developmental dyslexics; HF, high frequency; LF, low frequency; and RTs, reaction times.

Results for the GLMM on word RTs are displayed in Figure 1. Significant main effects were observed for Group, *F*(1,34) = 17.54, *p* < 0.001, Length, *F*(2,168) = 21.98, *p* < 0.001, and Frequency, *F*(1,168) = 79.85, *p* < 0.001. Significant interactions were observed for Group × Length × Frequency, *F*(2,5877) = 15.83, *p* < 0.001, Group × Length, *F*(2,5877) = 56.30, *p* < 0.001, Group × Frequency, *F*(1,5877) = 144.50, *p* < 0.001, and Length × Frequency, *F*(2,168) = 8.93, *p* < 0.001. The results of this word reading task demonstrate that only the DD group was affected by length and this effect was larger for longer unfamiliar words, particularly in the low frequency condition between lengths three and seven (*t* = −8.28, *p* < 0.001) and lengths five and seven (*t* = −7.67, *p* < 0.001). No LEs were present in the high frequency condition for the DD group (*p*s ≥ 0.908). The TDR group did not show any LEs (*p*s ≥ 0.980). *Post hoc* analyses on the three-way interaction are presented in Table 2.

**TABLE 2:** Word reading *post hoc* comparisons on the raw data using Tukey correction.

	**Contrast**	**Estimate**	**SE**	***t* ratio**	***p* value**
1	HF,3,TDR – LF,3,TDR	–0.30	44.09	–0.01	1.000
2	HF,3,TDR – HF,5,TDR	13.11	44.09	0.30	1.000
3	HF,3,TDR – LF,5,TDR	–25.38	44.09	–0.58	1.000
4	HF,3,TDR – HF,7,TDR	25.56	44.09	0.58	1.000
5	HF,3,TDR – LF,7,TDR	–31.45	44.09	–0.71	1.000
6	HF,3,TDR – HF,3,DD	–118.68	35.60	–3.33	0.041
7	HF,3,TDR – LF,3,DD	–300.78	44.09	–6.82	0.000
8	HF,3,TDR – HF,5,DD	–162.28	44.09	–3.68	0.014
9	HF,3,TDR – LF,5,DD	–327.70	44.09	–7.43	0.000
10	HF,3,TDR – HF,7,DD	–189.30	44.09	–4.29	0.001
11	HF,3,TDR – LF,7,DD	–666.01	44.09	–15.11	0.000
12	LF,3,TDR – HF,5,TDR	13.40	44.09	0.30	1.000
13	LF,3,TDR – LF,5,TDR	–25.08	44.09	–0.57	1.000
14	LF,3,TDR – HF,7,TDR	25.86	44.09	0.59	1.000
15	LF,3,TDR – LF,7,TDR	–31.16	44.09	–0.71	1.000
16	LF,3,TDR – HF,3,DD	–118.38	44.09	–2.69	0.238
17	LF,3,TDR – LF,3,DD	–300.48	35.60	–8.44	0.000
18	LF,3,TDR – HF,5,DD	–161.98	44.09	–3.67	0.014
19	LF,3,TDR – LF,5,DD	–327.41	44.09	–7.43	0.000
20	LF,3,TDR – HF,7,DD	–189.00	44.09	–4.29	0.001
21	LF,3,TDR – LF,7,DD	–665.72	44.09	–15.10	0.000
22	HF,5,TDR – LF,5,TDR	–38.49	44.09	–0.87	0.999
23	HF,5,TDR – HF,7,TDR	12.45	44.09	0.28	1.000
24	HF,5,TDR – LF,7,TDR	–44.56	44.09	–1.01	0.997
25	HF,5,TDR – HF,3,DD	–131.78	44.09	–2.99	0.116
26	HF,5,TDR – LF,3,DD	–313.89	44.09	–7.12	0.000
27	HF,5,TDR – HF,5,DD	–175.39	35.60	–4.93	0.000
28	HF,5,TDR – LF,5,DD	–340.81	44.09	–7.73	0.000
29	HF,5,TDR – HF,7,DD	–202.41	44.09	–4.59	0.000
30	HF,5,TDR – LF,7,DD	–679.12	44.09	–15.40	0.000
31	LF,5,TDR –HF,7,TDR	50.94	44.09	1.16	0.992
32	LF,5,TDR – LF,7,TDR	–6.07	44.09	–0.14	1.000
33	LF,5,TDR – HF,3,DD	–93.30	44.09	–2.12	0.611
34	LF,5,TDR – LF,3,DD	–275.40	44.09	–6.25	0.000
35	LF,5,TDR – HF,5,DD	–136.90	44.09	–3.11	0.085
36	LF,5,TDR – LF,5,DD	–302.33	35.60	–8.49	0.000
37	LF,5,TDR – HF,7,DD	–163.92	44.09	–3.72	0.012
38	LF,5,TDR – LF,7,DD	–640.64	44.09	–14.53	0.000
39	HF,7,TDR – LF,7,TDR	–57.01	44.09	–1.29	0.980
40	HF,7,TDR – HF,3,DD	–144.24	44.09	–3.27	0.053
41	HF,7,TDR – LF,3,DD	–326.34	44.09	–7.40	0.000
42	HF,7,TDR – HF,5,DD	–187.84	44.09	–4.26	0.002
43	HF,7,TDR – LF,5,DD	–353.26	44.09	–8.01	0.000
44	HF,7,TDR – HF,7,DD	–214.86	35.60	–6.03	0.000
45	HF,7,TDR – LF,7,DD	–691.57	44.09	–15.69	0.000
46	LF,7,TDR – HF,3,DD	–87.22	44.09	–1.98	0.708
47	LF,7,TDR – LF,3,DD	–269.33	44.09	–6.11	0.000
48	LF,7,TDR – HF,5,DD	–130.83	44.09	–2.97	0.122
49	LF,7,TDR – LF,5,DD	–296.25	44.09	–6.72	0.000
50	LF,7,TDR – HF,7,DD	–157.85	44.09	–3.58	0.020
51	LF,7,TDR – LF,7,DD	–634.56	35.60	–17.82	0.000
52	HF,3,DD – LF,3,DD	–182.10	44.09	–4.13	0.003
53	HF,3,DD – HF,5,DD	–43.60	44.09	–0.99	0.998
54	HF,3,DD – LF,5,DD	–209.03	44.09	–4.74	0.000
55	HF,3,DD – HF,7,DD	–70.62	44.09	–1.60	0.908
56	HF,3,DD – LF,7,DD	–547.34	44.09	–12.41	0.000
57	LF,3,DD – HF,5,DD	138.50	44.09	3.14	0.077
58	LF,3,DD – LF,5,DD	–26.92	44.09	–0.61	1.000
59	LF,3,DD – HF,7,DD	111.48	44.09	2.53	0.325
60	LF,3,DD – LF,7,DD	–365.23	44.09	–8.28	0.000
61	HF,5,DD – LF,5,DD	–165.42	44.09	–3.75	0.011
62	HF,5,DD – HF,7,DD	–27.02	44.09	–0.61	1.000
63	HF,5,DD – LF,7,DD	–503.73	44.09	–11.43	0.000
64	LF,5,DD – HF,7,DD	138.41	44.09	3.14	0.077
65	LF,5,DD – LF,7,DD	–338.31	44.09	–7.67	0.000
66	HF,7,DD – LF,7,DD	–476.71	44.09	–10.81	0.000

*TDR, typically developing readers; DD, developmental dyslexics; HF, high frequency; and LF, low frequency.*

#### *Z*-Scores

**FIGURE 2:**
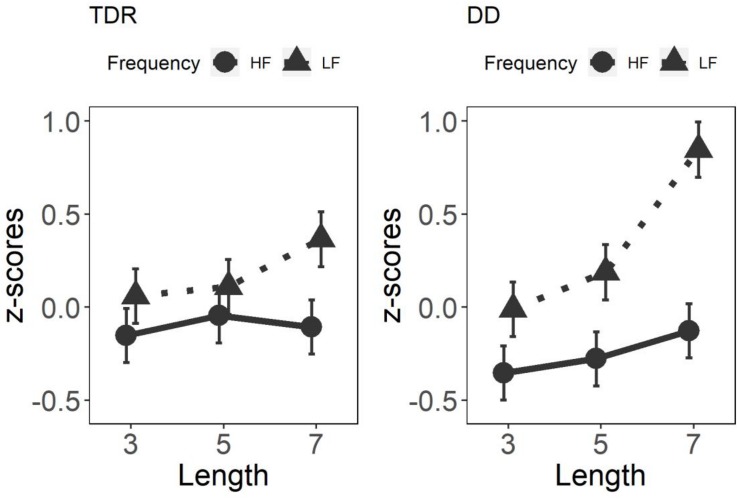
Three-way interaction on *z*-scores on words. Higher *z*-scores reflect lower performance. TDR, typical developing readers; DD, developmental dyslexics; HF, high frequency; and LF, low frequency.

Results for the GLMM on word *z*-scores are displayed in Figure 2. Significant main effects were observed for Length, *F*(2,165) = 14.07, *p* < 0.001, and Frequency, *F*(1,165) = 59.37, *p* < 0.001, with no effect of Group, *F*(1,5905) = 0.08, *p* = 0.779. This latter result is not surprising since all individual performances have been centered to the zero through the *z*-score transformation. Significant interactions were observed for Group × Length × Frequency, *F*(2,5905) = 5.76, *p* < 0.001, Group × Length, *F*(2,5905) = 25.66, *p* < 0.001, Group × Frequency, *F*(1,5905) = 49.13, *p* < 0.001, and Length × Frequency, *F*(2,165) = 6.38, *p* < 0.001. The results obtained with the *z*-score transformation replicated those obtained with the raw data. *Post hoc* analyses on the three-way interaction are presented in Table 3.

**TABLE 3:** Word reading *post hoc* comparisons on the *z*-scores using Tukey correction.

	**Contrast**	**Estimate**	**SE**	***t* ratio**	***p* value**
1	HF,3,TDR – LF,3,TDR	–0.21	0.11	–1.94	0.736
2	HF,3,TDR – HF,5,TDR	–0.10	0.11	–0.92	0.999
3	HF,3,TDR – LF,5,TDR	–0.27	0.11	–2.53	0.329
4	HF,3,TDR – HF,7,TDR	–0.05	0.11	–0.50	1.000
5	HF,3,TDR – LF,7,TDR	–0.51	0.11	–4.80	0.000
6	HF,3,TDR – HF,3,DD	0.19	0.06	3.44	0.029
7	HF,3,TDR – LF,3,DD	–0.16	0.11	–1.52	0.932
8	HF,3,TDR – HF,5,DD	0.11	0.11	1.01	0.997
9	HF,3,TDR – LF,5,DD	–0.36	0.11	–3.35	0.044
10	HF,3,TDR – HF,7,DD	–0.05	0.11	–0.45	1.000
11	HF,3,TDR – LF,7,DD	–1.01	0.11	–9.33	0.000
12	LF,3,TDR – HF,5,TDR	0.11	0.11	1.01	0.997
13	LF,3,TDR – LF,5,TDR	–0.06	0.11	–0.60	1.000
14	LF,3,TDR – HF,7,TDR	0.15	0.11	1.44	0.955
15	LF,3,TDR – LF,7,TDR	–0.31	0.11	–2.87	0.159
16	LF,3,TDR – HF,3,DD	0.40	0.11	3.71	0.014
17	LF,3,TDR – LF,3,DD	0.04	0.06	0.77	1.000
18	LF,3,TDR – HF,5,DD	0.31	0.11	2.95	0.133
19	LF,3,TDR – LF,5,DD	–0.16	0.11	–1.44	0.955
20	LF,3,TDR – HF,7,DD	0.16	0.11	1.49	0.942
21	LF,3,TDR – LF,7,DD	–0.80	0.11	–7.41	0.000
22	HF,5,TDR – LF,5,TDR	–0.17	0.11	–1.61	0.903
23	HF,5,TDR – HF,7,TDR	0.05	0.11	0.42	1.000
24	HF,5,TDR – LF,7,TDR	–0.42	0.11	–3.88	0.007
25	HF,5,TDR – HF,3,DD	0.29	0.11	2.70	0.231
26	HF,5,TDR – LF,3,DD	–0.06	0.11	–0.61	1.000
27	HF,5,TDR – HF,5,DD	0.21	0.06	3.75	0.010
28	HF,5,TDR – LF,5,DD	–0.26	0.11	–2.44	0.384
29	HF,5,TDR – HF,7,DD	0.05	0.11	0.48	1.000
30	HF,5,TDR – LF,7,DD	–0.91	0.11	–8.43	0.000
31	LF,5,TDR – HF,7,TDR	0.22	0.11	2.03	0.671
32	LF,5,TDR – LF,7,TDR	–0.24	0.11	–2.25	0.515
33	LF,5,TDR – HF,3,DD	0.46	0.11	4.29	0.002
34	LF,5,TDR – LF,3,DD	0.11	0.11	1.00	0.998
35	LF,5,TDR – HF,5,DD	0.38	0.11	3.53	0.025
36	LF,5,TDR – LF,5,DD	–0.09	0.06	–1.56	0.923
37	LF,5,TDR – HF,7,DD	0.22	0.11	2.08	0.635
38	LF,5,TDR – LF,7,DD	–0.74	0.11	–6.77	0.000
39	HF,7,TDR – LF,7,TDR	–0.46	0.11	–4.30	0.001
40	HF,7,TDR – HF,3,DD	0.24	0.11	2.28	0.493
41	HF,7,TDR – LF,3,DD	–0.11	0.11	–1.03	0.997
42	HF,7,TDR – HF,5,DD	0.16	0.11	1.51	0.935
43	HF,7,TDR – LF,5,DD	–0.31	0.11	–2.86	0.164
44	HF,7,TDR – HF,7,DD	0.01	0.06	0.10	1.000
45	HF,7,TDR – LF,7,DD	–0.96	0.11	–8.85	0.000
46	LF,7,TDR – HF,3,DD	0.70	0.11	6.57	0.000
47	LF,7,TDR – LF,3,DD	0.35	0.11	3.26	0.057
48	LF,7,TDR – HF,5,DD	0.62	0.11	5.80	0.000
49	LF,7,TDR – LF,5,DD	0.15	0.11	1.40	0.962
50	LF,7,TDR – HF,7,DD	0.47	0.11	4.35	0.001
51	LF,7,TDR – LF,7,DD	–0.49	0.06	–8.42	0.000
52	HF,3,DD – LF,3,DD	–0.35	0.11	–3.30	0.051
53	HF,3,DD – HF,5,DD	–0.08	0.11	–0.77	1.000
54	HF,3,DD – LF,5,DD	–0.55	0.11	–5.10	0.000
55	HF,3,DD – HF,7,DD	–0.24	0.11	–2.23	0.533
56	HF,3,DD – LF,7,DD	–1.20	0.11	–11.09	0.000
57	LF,3,DD – HF,5,DD	0.27	0.11	2.53	0.326
58	LF,3,DD – LF,5,DD	–0.20	0.11	–1.83	0.801
59	LF,3,DD – HF,7,DD	0.12	0.11	1.08	0.995
60	LF,3,DD – LF,7,DD	–0.85	0.11	–7.78	0.000
61	HF,5,DD – LF,5,DD	–0.47	0.11	–4.34	0.001
62	HF,5,DD – HF,7,DD	–0.16	0.11	–1.46	0.950
63	HF,5,DD – LF,7,DD	–1.12	0.11	–10.33	0.000
64	LF,5,DD – HF,7,DD	0.31	0.11	2.91	0.146
65	LF,5,DD – LF,7,DD	–0.65	0.11	–5.90	0.000
66	HF,7,DD – LF,7,DD	–0.96	0.11	–8.89	0.000

*TDR, typically developing readers; DD, developmental dyslexics; HF, high frequency; and LF, low frequency.*

#### Errors

**FIGURE 3:**
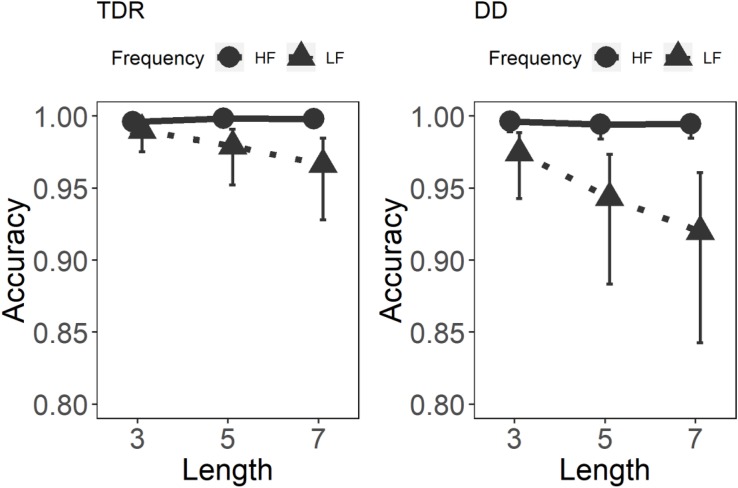
Error rates in the two groups in each individual condition. TDR, typical developing readers; DD, developmental dyslexics; HF, high frequency; and LF, low frequency.

Results for the GLMM on word errors are displayed in Table 1 and Figure 3. Significant main effects were observed for Group, *z* = −2.73, *p* = 0.006, and Frequency, *z* = −7.22, *p* < 0.001. For Length, only the difference between lengths three and seven was significant, *z* = −2.12, *p* < 0.05. These results demonstrate that the DD group performed worse than the TDR group. Additionally, both groups were more accurate in the high frequency condition as shown by the main effect of frequency. Intriguingly, the performance in both groups was very high. Only the longest words (7 letters) were read worse than the other words in the DD group.

### Non-word Reading

#### Reaction Times

**FIGURE 4:**
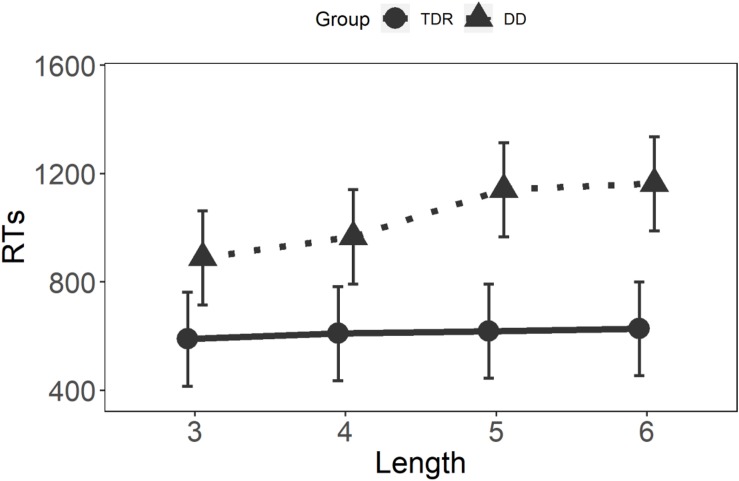
Two-way interaction on non-words. TDR, typical developing readers; DD, developmental dyslexics; and RTs, reaction times.

Results for the GLMM on non-word RTs are displayed in Figure 4. Significant main effects were observed for Group, *F*(1,34) = 12.60, *p* < 0.001, and Length, *F*(3,63) = 12.52, *p* < 0.001. A significant interaction was observed for Group × Length, *F*(3,2132) = 16.20, *p* < 0.001. The results of this non-word reading task demonstrate that the DD group was affected by non-word length, with significant differences between lengths three and five (*t* = −6.80, *p* < 0.001), lengths three and six (*t* = −7.48, *p* < 0.001), lengths four and five (*t* = −4.70, *p* < 0.001), and length four and six (*t* = −5.35, *p* < 0.001). No differences were present between length three and four (*p* = 0.413). The TDR group did not show any LEs (*p* ≥ 0.962). *Post hoc* analyses on the interaction are presented in Table 4.

**TABLE 4:** Non-words *post hoc* comparisons on the raw data using Tukey correction.

	**Contrast**	**Estimate**	**SE**	***t* ratio**	***p* value**
1	3,TDR – 4,TDR	–19.98	35.77	–0.56	0.999
2	3,TDR – 5,TDR	–29.27	35.82	–0.82	0.992
3	3,TDR – 6,TDR	–38.17	35.78	–1.07	0.962
4	3,TDR – 3,DD	–299.85	123.35	–2.43	0.257
5	3,TDR – 4,DD	–377.27	125.21	–3.01	0.078
6	3,TDR – 5,DD	–551.27	125.31	–4.40	0.002
7	3,TDR – 6,DD	–573.52	125.19	–4.58	0.001
8	4,TDR – 5,TDR	–9.30	35.72	–0.26	1.000
9	4,TDR – 6,TDR	–18.19	35.68	–0.51	1.000
10	4,TDR – 3,DD	–279.87	125.17	–2.24	0.354
11	4,TDR – 4,DD	–357.29	123.36	–2.90	0.102
12	4,TDR – 5,DD	–531.29	125.28	–4.24	0.003
13	4,TDR – 6,DD	–553.54	125.16	–4.42	0.002
14	5,TDR – 6,TDR	–8.90	35.73	–0.25	1.000
15	5,TDR – 3,DD	–270.58	125.18	–2.16	0.396
16	5,TDR – 4,DD	–347.99	125.20	–2.78	0.130
17	5,TDR – 5,DD	–522.00	123.46	–4.23	0.003
18	5,TDR – 6,DD	–544.24	125.18	–4.35	0.002
19	6,TDR – 3,DD	–261.68	125.17	–2.09	0.438
20	6,TDR – 4,DD	–339.09	125.19	–2.71	0.150
21	6,TDR – 5,DD	–513.10	125.29	–4.10	0.005
22	6,TDR – 6,DD	–535.34	123.34	–4.34	0.002
23	3,DD – 4,DD	–77.41	36.68	–2.11	0.413
24	3,DD – 5,DD	–251.42	36.99	–6.80	0.000
25	3,DD – 6,DD	–273.66	36.59	–7.48	0.000
26	4,DD – 5,DD	–174.01	37.05	–4.70	0.000
27	4,DD – 6,DD	–196.25	36.65	–5.35	0.000
28	5,DD – 6,DD	–22.24	36.95	–0.60	0.999

*TDR, typically developing readers and DD, developmental dyslexics.*

#### *Z*-Scores

**FIGURE 5:**
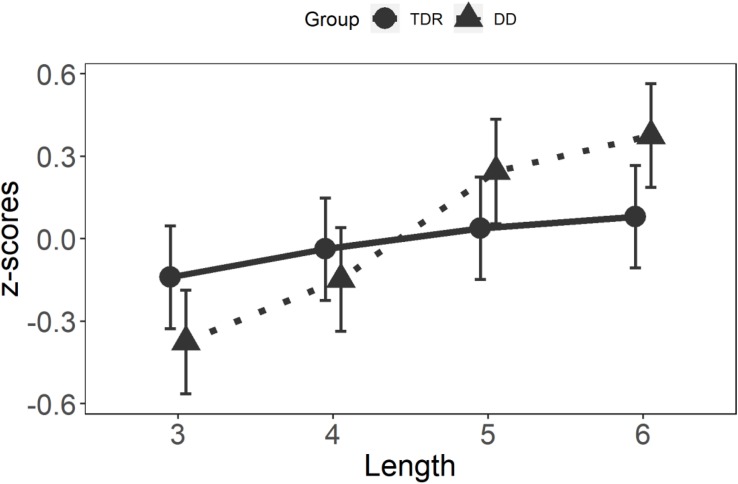
Two-way interaction on *z*-scores on non-words. Higher *z*-scores reflect lower performance. TDR, typical developing readers and DD, developmental dyslexics.

Results for the GLMM on non-word *z*-scores are displayed in Figure 5. A significant main effect was observed for Length, *F*(3,63) = 6.21, *p* < 0.001, with no effect of Group, *F*(1,2160) = 1.19, *p* = 0.276. This latter result is not surprising since all individual performances have been centered to the zero through the *z*-score transformation. A significant interaction was observed for Group × Length, *F*(3,2160) = 12.32, *p* < 0.001. These results confirmed those obtained with the raw data. *Post hoc* analyses on the interaction are presented in Table 5.

**TABLE 5:** Non-words *post hoc* comparisons on the *z*-scores using Tukey correction.

	**Contrast**	**Estimate**	**SE**	***t* ratio**	***p* value**
1	3,TDR – 4,TDR	–0.10	0.13	–0.76	0.994
2	3,TDR – 5,TDR	–0.18	0.13	–1.33	0.886
3	3,TDR – 6,TDR	–0.22	0.13	–1.64	0.726
4	3,TDR – 3,DD	0.23	0.07	3.27	0.024
5	3,TDR – 4,DD	0.01	0.14	0.05	1.000
6	3,TDR – 5,DD	–0.38	0.14	–2.83	0.101
7	3,TDR – 6,DD	–0.52	0.14	–3.81	0.006
8	4,TDR – 5,TDR	–0.08	0.13	–0.56	0.999
9	4,TDR – 6,TDR	–0.12	0.13	–0.87	0.988
10	4,TDR – 3,DD	0.34	0.14	2.49	0.212
11	4,TDR – 4,DD	0.11	0.07	1.54	0.788
12	4,TDR – 5,DD	–0.28	0.14	–2.08	0.438
13	4,TDR – 6,DD	–0.41	0.14	–3.05	0.058
14	5,TDR – 6,TDR	–0.04	0.13	–0.31	1.000
15	5,TDR – 3,DD	0.41	0.14	3.05	0.058
16	5,TDR – 4,DD	0.19	0.14	1.37	0.867
17	5,TDR – 5,DD	–0.21	0.07	–2.83	0.087
18	5,TDR – 6,DD	–0.34	0.14	–2.49	0.213
19	6,TDR – 3,DD	0.45	0.14	3.36	0.024
20	6,TDR – 4,DD	0.23	0.14	1.68	0.698
21	6,TDR – 5,DD	–0.16	0.14	–1.21	0.927
22	6,TDR – 6,DD	–0.30	0.07	–4.13	0.001
23	3,DD – 4,DD	–0.23	0.14	–1.67	0.707
24	3,DD – 5,DD	–0.62	0.14	–4.53	0.000
25	3,DD – 6,DD	–0.75	0.14	–5.52	0.000
26	4,DD – 5,DD	–0.39	0.14	–2.87	0.091
27	4,DD – 6,DD	–0.52	0.14	–3.84	0.005
28	5,DD – 6,DD	–0.13	0.14	–0.96	0.979

*TDR, typically developing readers and DD, developmental dyslexics.*

#### Errors

Results for the GLMM on non-word errors are displayed in Table 1. A significant main effect was observed for group only, Group, *z* = −3.03, *p* = 0.002, reflecting the fact that the TDR group was more accurate than the DD group.

## Discussion

The aim of this study was to investigate whether the effect of word length, usually investigated in adult DD readers of a transparent orthography, may also characterize the reading of English individuals with DD. In this study, we wanted to verify whether participants with DD showed an over reliance on the sub-lexical route, with a consequent increase in the time needed to read words and non-words of increasing length (i.e., LE). For this reason, we compared a group of participants with DD to a group of TDRs in word and non-word reading tasks.

The results of this study indicate that participants with DD did indeed present with a strong LE, compared to TDRs, in both word and non-word reading, which was particularly evident in RTs. The DD group showed a marked decrease in speed of reading as a function of the number of letters in a word. These results are similar to those observed with adult participants in transparent orthographies (Zoccolotti et al., 2005; Davies et al., 2007; Richlan et al., 2010; Suárez-Coalla and Cuetos, 2015) and with children reading English (Ziegler et al., 2003). A possible explanation for these results may be that participants in the DD group predominantly rely on a serial analysis of the item, remaining anchored to a sub-lexical reading strategy, which results in slower and more effortful reading. For the word reading task, intriguingly, the marked differences in the DD group were in low frequency words, particularly between length three and length seven and between length five and length seven, whereas no statistically significant differences were found between different lengths in the high frequency condition, as shown by the *post hoc* comparisons (see Table 2). These results may indicate that the DD group employed larger units to read familiar words whereas, they appear to switch to smaller units when reading longer unfamiliar words.

The use of larger and smaller units in reading is postulated by the grain size theory (Ziegler and Goswami, 2005). The grain size hypothesis assumes that readers of inconsistent orthographies rely to a greater extent on larger units or grain sizes (e.g., syllables or even whole words), whereas readers of more consistent orthographies such as Italian, tend to rely on smaller grain sizes (e.g., graphemes) with the reading output primarily based on grapheme-phoneme correspondence. That is, the opaquer the orthography, the larger the units employed in reading. Participants with DD were affected by the frequency of the words with familiar words being read better than unfamiliar words at each length considered. This pattern is consistent with the employment of a lexical route by the DD group to read familiar words. These findings were confirmed by the *z*-score analyses and mirrored those found with adult DDs reading in a transparent orthography (see e.g., Yael et al., 2015).

Aspects of the TDR group performance are also interesting to note. In contrast to earlier studies (e.g., Balota et al., 2004), we did not find any significant LE for words or non-words. Our results fit well with previous research where LE has not been found among adult English readers, except in studies which employ a large number of items and lengths (see Marinelli et al., 2016 on this point). However, the results obtained with the *z*-scores showed that low frequency seven letter words differed from the other lengths. This result may indicate that the TDR group struggle to read long, unfamiliar words, and hence the TDR performance might be affected by the length of the words.

Intriguingly, the TDR group did not show any advantage in reading high frequency words compared to low frequency words (i.e., frequency effect). We can speculate that the employment of larger units by the TDR group might determine the almost total absence of advantage in reading high frequency words compared to low frequency words. In fact, even if a difference is noticeable in terms of means in RTs between low frequency and high frequency words, such difference is not statistically significant, except in the case of the seven letter low frequency condition and only in the *z*-scores (see Table 3). Nevertheless, it is worth noting that this result might be due to the effects of the transformation in *z*-scores.

Overall the results obtained from the *z*-score transformation are consistent with those obtained using the RTs. However, it is worth stating that in this particular case *z*-score transformation might be somewhat problematic. It has been argued that to the extent that the product of intrinsic variability and processing rate differs across individuals, the *z*-score transformation will be differentially biased for individuals (Faust et al., 1999). In this study, we found that the variability in the TDR group was much smaller, compared to the variability in the DD group. Therefore, when the raw scores are transformed to *z*-scores in the TDR group, even very small differences tend to be magnified. Such an effect seems to reflect more differences in the variance than an intrinsic difference between the two groups.

Typically developing readers seem to read familiar words by directly accessing the orthographic representation of the word (whole word recognition strategy) and unfamiliar words through the employment of large chunks such as the pattern of letters, syllables or rimes (e.g., Brown and Deavers, 1999). As previously illustrated, the inconsistency of English, in which the correspondence between letters and sounds is not always predictable, leads readers of this orthography to rely on a larger grain size to read. Indeed, the employment of smaller grain sizes by English readers is more likely to result in errors. The present results are therefore consistent with previous accounts of the use of larger units and a parallel processing mode in English readers (Ziegler and Goswami, 2005; Marinelli et al., 2016). Furthermore, the use of larger units in this group seems to help them to read fast even unfamiliar words, showing a minimum and not statistically significant frequency effect. DD participants, instead, seem to employ smaller grain sizes to read longer and unfamiliar words, which in turn cause an increase in the response latency and the LE. However, the frequency effect showed by such participants seems to highlight that they are still able to employ a parallel processing of the words when they are familiar.

Some useful insight can also be drawn by considering accuracy rates. Both groups were more accurate in reading high than low frequency words. This frequency effect shown by DDs also in RTs confirms the availability of the lexical route in the DD group (Barca et al., 2006). Furthermore, the largest number of errors for both groups was in the low frequency set of five and seven letter lengths. This reflects the fact that in an opaque orthography, like English, long unfamiliar words might be more difficult to read than familiar words even for proficient readers, increasing the amount of errors.

The non-word reading task, employed to investigate sub-lexical decoding, showed that LE in RTs were more apparent in the DD group, than in the TDR group. The marked differences in the DD group were detected between shorter non-words and longer non-words. Indeed, no significant LE was found between three letter and four letter non-words, whereas a difference was found between three letter and five letter, three letter and six letter, four letter and five letter and four letter and six letter non-words. These results confirm that DDs can employ larger grain sizes to read even shorter non-words. However, increasing the number of letters results in smaller grain sizes being employed.

Interestingly, the TDR group did not show any LE in the non-word task, confirming that the employment of larger grain sizes is the prevailing way to read in this group, even when they encounter unfamiliar words. Indeed, the absence of a LE in the TDR group in this task is entirely consistent with the employment of larger grain sizes in typical readers of opaque orthographies compared to transparent orthographies. As for the accuracy data, the DD group made more errors than the TDR group, whose performance was also high in this task. The results obtained with the raw data were replicated with the *z*-scores, demonstrating that these findings are robust and might indicate that the DD group struggled with the sub-lexical decoding.

Overall, these findings suggest that the DD group presents with a large LE in both word and non-word reading, compared to TDRs, who showed very little difference between conditions in all the measures and tasks considered. Although this result seems to point to a deficit of the lexical route and an over-reliance on the sub-lexical route in DD, the frequency effect shown by DDs allows us to speculate that the lexical route is still available to this group. Furthermore, the difficulties shown by DDs in the non-word reading point out that they also struggle in the sub-lexical decoding. In terms of the DRC model, it is possible that the difficulties in DD arise at an earlier stage of the model, in particular at the visual feature or at the letter unit system.

An alternative explanation of the findings comes from studies conducted with patients with pure alexia. As previously mentioned, these patients present with damage to the left fusiform gyrus in the ventral occipito-temporal cortex, an area known as the visual word form area (Dehaene and Cohen, 2011). This area seems to be involved in pre-lexical processing of visual word forms (e.g., Dehaene et al., 2005). Behaviorally, pure alexia is characterized by a slowing of letter/word processing with some participants only able to read words by identifying one letter at a time. Using sensitive non-orthographic visual tests (naming line drawings of objects, novel face matching, checkerboard and kanji character discrimination), these patients also show deficits in pattern discrimination, object naming, and face processing, and are slower as a function of the visual complexity of the stimuli (Roberts et al., 2013, 2015; Woollams et al., 2014). Future research should then investigate whether participants with DD also present with deficits in non-orthographic visual processing using the same tasks (i.e., checkerboard discrimination, novel face matching). If so, the triangle model (Patterson and Lambon Ralph, 1999; Hoffman et al., 2015) might be a more parsimonious account of these results than the DRC model and the application of the domain-general cognitive neuropsychological approach in explaining DD may prove valuable.

Establishing which model best accounts fits our findings is, however, is beyond the scope of this paper. Nevertheless, it would be useful for future studies to test participants with DD on the visual tasks mentioned above, work which we have already begun (Provazza et al., 2019). This would seem to be particularly relevant since patients with pure alexia present with LEs associated with other visual impairments (e.g., Roberts et al., 2013). Furthermore, similar brain abnormalities (e.g., left vOT) have been noted in DD using different methods including total brain volume, voxel- and surface- based morphometry, white matter, diffusion imaging, brain gyrification, and tissue metabolite (for review see Ramus et al., 2018). Consequently, an association seems to exist between the neural bases of dyslexia (acquired and developmental) and visual and phonological impairments. It would also be interesting to compare participants with DD reading different orthographies such as Italian and English (transparent vs. opaque; see Marinelli et al., 2016 on this point).

To summarize, our results have shown that the LE seems to characterize DD not only in transparent but also in opaque orthographies, like English. This research presents an original contribution to our understanding of DD in English speakers. In fact, in the extant literature, LEs appear to be scarcely evaluated in DD in opaque orthographies and, in particular, in adults with DD. Furthermore, this study clearly showed that participants with DD are severely impaired in RTs, whereas they performed better in terms of accuracy, although this was lower compared to that of the TDR group.

It is worth noting that this study presents with some limitations. For instance, participants have not been matched for IQ. However, we would expect differences in IQ to be insignificant in this sample of academically able adults in higher education and thus would not impact substantially the conclusions drawn. IQ is a very generic and broad concept, and in fact, the use of some intelligence batteries has been recently questioned. For example, some authors (Giofrè and Cornoldi, 2015; Giofrè et al., 2019) have highlighted important biases in the use of intelligence estimates in studies of children with learning disabilities. Principally, differences in IQs might reflect artifacts of the battery in use, rather than real differences in the proposed latent variables. Notwithstanding the conclusions drawn from the present sample, we do acknowledge that perhaps in more differentiated samples the use of intelligence tests, may be worthwhile (see e.g., Kemp et al., 2009; Paizi et al., 2013). A further limitation might be the sample size, which was not very large. Nevertheless, the *a priori* power analysis showed that a sample size of 36 participants was sufficient to obtain robust results. Moreover, the analytic approach that we employed (i.e., generalized linear mixed models), strengthened the experimental power of the by-subject and by-item analyses and limited the loss of information due to the prior averaging of the by-subject and by-item analyses (Baayen et al., 2002; Paizi et al., 2013). Despite these limitations, the results of this study provide insight into LEs in adult participants with DD reading in an opaque orthography and show that the LE is a critical feature in DD regardless of the orthography. Additionally, since LEs are observed in highly educated participants with DD, it might be an aspect to be clinically assessed in adults with DD in higher education and beyond. Previous research indeed has shown a lack of consensus about how university students should be diagnosed, since their performance in achievement tests is often in the average range (e.g., Sparks and Lovett, 2009). These findings might prove fruitful to clinicians working with DD university students, although further research is needed to confirm the results obtained in this study.

## Data Availability Statement

The datasets generated for this study are available on request to the corresponding author.

## Ethics Statement

The studies involving human participants were reviewed and approved by the Liverpool John Moores University Research Ethics Committee. The patients/participants provided their written informed consent to participate in this study.

## Author Contributions

All authors contributed to the study design, drafted the manuscript, provided the critical revisions, and approved the final version of the manuscript for submission. SP performed the test and data collection. SP, DG, and DR performed the data analysis and interpretation.

## Conflict of Interest

The authors declare that the research was conducted in the absence of any commercial or financial relationships that could be construed as a potential conflict of interest.
